# Influence of interface dielectric disorder on interlayer excitons in mixed binary/ternary TMD heterostructures†

**DOI:** 10.1039/d4na00786g

**Published:** 2025-07-25

**Authors:** Mohammed Adel Aly, Emmanuel Oghenevo Enakerakpor, Hilary Masenda, Martin Koch

**Affiliations:** a Faculty of Physics and Materials Sciences Center, Philipps-Universität Marburg 35032 Marburg Germany martin.koch@physik.uni-marburg.de mohammed.nouh@physik.uni-marburg.de; b Department of Physics, Faculty of Science, Ain Shams University 11566 Cairo Egypt; c School of Physics, University of the Witwatersrand 2050 Johannesburg South Africa hilary.masenda@wits.ac.za

## Abstract

In this paper, we study the excitonic linewidths and peak energies in two transition metal dichalcogenide heterostructures of Mo_0.5_W_0.5_Se_2_ and its binary counterparts, MoSe_2_ and WSe_2_. We observe spectra composed of several individual excitonic transitions in temperature-dependent photoluminescence measurements. Among these are transitions of neutral excitons and trions from the binary layers and the interlayer excitons from the heterostructures. The luminescence linewidth of the interlayer excitons is significantly broader than the linewidths of the excitonic transitions from the binary layers. We attribute this additional line broadening to dielectric disorder caused by spatial inhomogeneity at the interface.

## Abstract 2

Interlayer excitons in binary–ternary heterostructures show much broader linewidths than those in intralayer binary monolayers. A phenomenon is attributed to dielectric disorder caused by interface inhomogeneities and alloy disorder.

## Graphical abstract

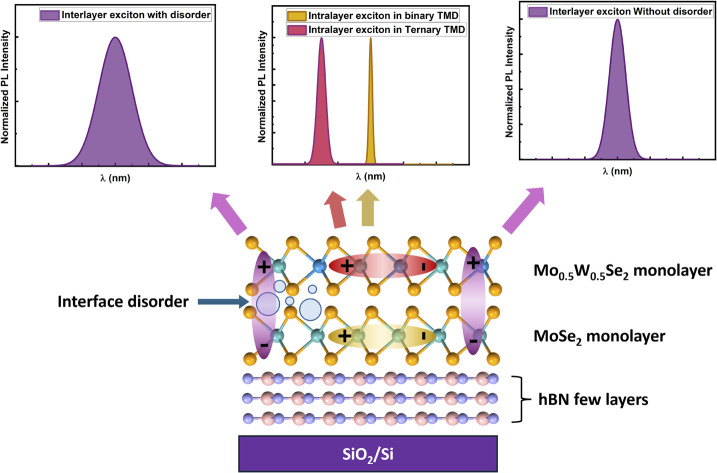

## 1 Introduction

Transition metal dichalcogenides (TMDs) as a class of materials have received much attention in recent years, especially in the form of monolayer thin films.^1–9^ Proposed devices based on TMDs include circuits with gate controllable exciton transport, vertical field-effect transistors, infrared photodetectors, and spin-filtering devices.^10–12^ Recently, a large-scale integrated vector–matrix multiplication processor based on monolayer molybdenum disulfide memories was demonstrated.^13^

Typical representatives of this class of materials are Group VIB TMDs. They are indirect bandgap semiconductors in their bulk form, but become direct bandgap semiconductors when the film thickness drops to a monolayer.^1,14^ Of central importance in these materials are excitons. In TMDs, these bound electron–hole pairs have much higher binding energies than their counterparts in III–V semiconductors; a material class which has been used technologically for a long time.^15–19^ Studies on TMDs show that binding energy values for neutral excitons can vary widely from 320 to 720 meV,^3,20–31^ influenced by the surrounding dielectric environment. In contrast, GaAs and GaN quantum wells exhibit much lower binding energies for neutral excitons, typically between 6 and 30 meV.^32–35^ This difference highlights the unique electronic and optical properties of TMDs compared to III–V semiconductors. Due to the high binding energy, excitons in TMDs are still present at room temperature. Therefore, the Coulomb interaction must always be considered in the design of future TMD-based devices.

In theory, crystalline materials can be considered perfect, *i.e.* they can be perfectly ordered within their crystalline structure in a completely uniform dielectric environment. In reality, however, disorder plays a crucial role. In the case of TMDs, a monolayer can be largely perfect, *i.e.* have only a few lattice defects. However, the substrate and, hence, the dielectric environment can have unevenness and impurities. This type of disorder has already been reported for TMDs and is called dielectric disorder.^36^

Binary TMDs are the most extensively studied. Yet, of course, their alloys are also possible in the form of ternary or quaternary compounds with element mixing on either transition metal and chalcogen or both, respectively. In this case, alloy disorder is inevitable. This stems from the uneven random spatial distribution of elemental species. In III–V semiconductors, the impact of alloy disorder on the electronic and optical properties has already been studied intensively.^37–39^ The disorder then leads to the localization of electronic states as well as homogeneous and inhomogeneous line broadening. Besides, the magnitude of the Stokes shift depends on the energy scale of the disorder potential.^40^ Also, for TMD monolayers, the influence of alloy disorder on the excitonic properties has been studied.^41^

Almost all electronic and optoelectronic components are made up of several materials. Hence, they contain interfaces, which are pivotal in determining device performance. They influence charge carrier dynamics, energy band alignment, recombination processes, and quantum effects. Electrons or holes must pass across an interface when transferring between materials. For the operation of some devices like solar cells, the optically generated electrons and holes must be separated from each other, *i.e.* directed to move in different directions. A band structure alignment in which this occurs is called the type II structure.^42–44^ After charge separation, electrons and holes in two different materials still feel the Coulomb attraction to each other and form so-called charge-transfer states, and, if the temperature is low enough, they form charge transfer excitons (CTX); commonly referred to as interlayer excitons (ILX) in low-dimensional heterostructures. Only in extreme cases can ILX be observed in an absorption spectrum,^45,46^ usually they are observed only in the luminescence spectrum.^31,47,48^

**Fig. 1:**
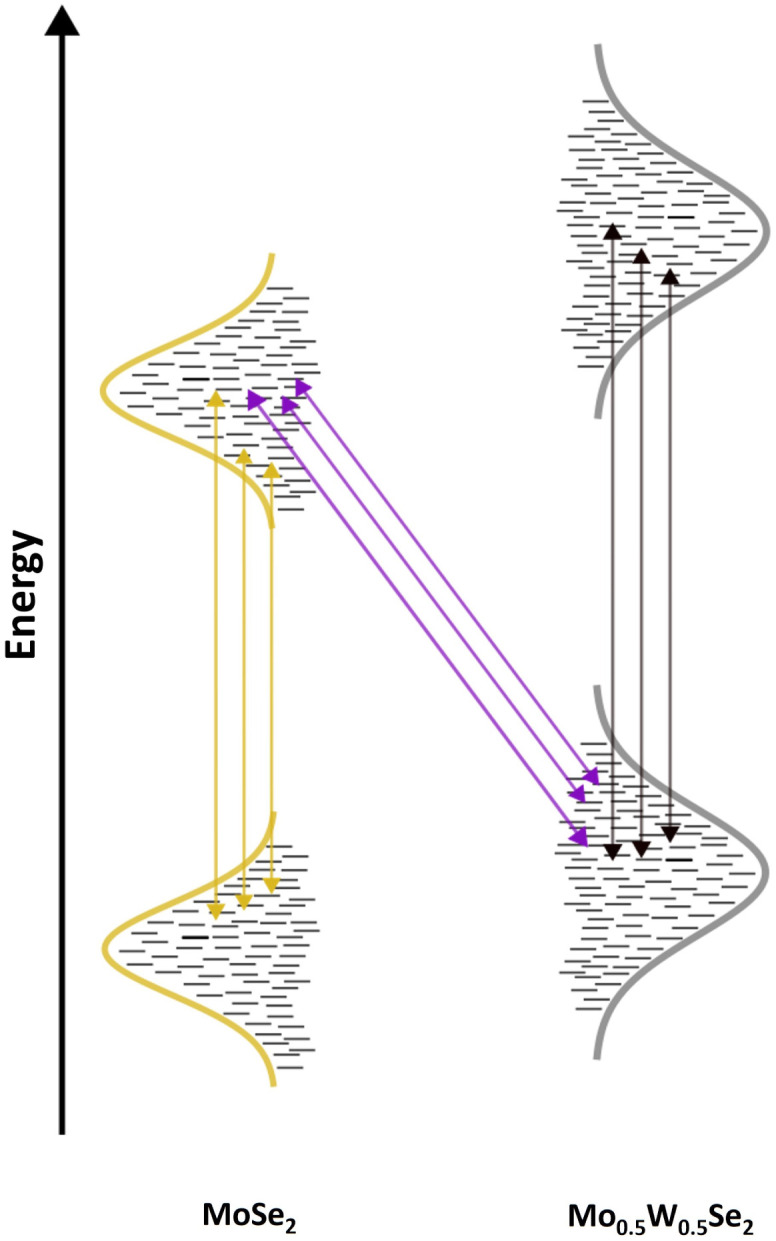
Schematic illustration of optical possible transitions in a Mo_0.5_W_0.5_Se_2_/MoSe_2_ heterostructure.

The question now is how alloy disorder affects the optical properties of these ILX in a mixed binary/ternary TMD heterostructure? What would we expect? In a binary semiconductor, thus also in a binary TMD monolayer, the optical band-to-band transition is not infinitely sharp but spectrally broadened. On the one hand, there is a homogeneous line broadening related to the so-called dephasing time.^49,50^ This homogeneous line broadening increases with temperature as the dephasing time decreases due to carrier–phonon scattering. Secondly, one has an inhomogeneous broadening due to material defects or dielectric disorder resulting from inhomogeneity of the substrate or an inhomogeneous coupling of the 2D layer to the substrate. Electronic states in the valence and conduction bands often follow a Gaussian distribution. Fig. 1 illustrates this schematically. Starting the discussion with the left portion, which shows the distribution of energy states in a binary 2D material. Energy variations in the valence and conduction bands are assumed to be connected. This means that if the conduction band states decrease, the valence band states will increase. The result is a distribution of transition energies indicated by the yellowish gold/dark gray vertical arrows in Fig. 1.

In this figure, the influence of the Coulomb interaction, which leads to excitonic states, is not yet considered. Recombination will typically occur out of the 1s exciton, further lowering the transition energies. Although disorder effects can also cause somewhat spatially varying excitonic binding energies, the picture that there is a distribution of transition energies over the sample area remains correct.

In the case of a ternary material, one has the same situation, but here the alloy disorder adds to the line broadening.^51–57^ This is indicated by the somewhat broader Gaussian distributions in the right part of Fig. 1. If a type II structure is present, as shown in Fig. 1, electrons and/or holes will be transferred to the neighbouring material layer. ILX are formed, which can subsequently decay radiatively. The oblique violet arrows indicate the recombination process that results from these states. In our schematic, the ILX forms from electron states in the binary material and hole states in the ternary material. However, the energetic broadening of these states forming the ILX is different. The energetic distributions of the resulting interlayer excitonic transitions in a binary–ternary heterostructure should be smaller than those of intralayer excitons in the ternary monolayer, where excitons form from two energetically broad electronic distributions due to the presence of compositional disorder. However, these interlayer energetic distributions will be broader than intralayer excitonic transitions in the binary monolayer, where the additional broadening of the electron and hole states is negligible. This stems from the absence of alloy disorder, which is present in the ternary system. Therefore, the broadening of the excitonic transition in the binary–ternary interlayer system will be less than that of intralayer excitonic transitions in the ternary monolayer but higher than that of intralayer excitonic transitions in the binary monolayer. In other words, the inhomogeneous linewidth ILX in a heterostructure of TMD monolayers, where one monolayer contains a ternary material, should be in between the inhomogeneous linewidth of the binary material and that of the ternary material.

In this paper, we investigate if this expectation can be confirmed experimentally. Studies on binary–ternary heterobilayers from Mo_0.5_W_0.5_Se_2_ and its binary counterparts, MoSe_2_ and WSe_2_ were undertaken. The samples were fabricated by micro-mechanical exfoliation followed by a dry transfer and stacking technique. We perform temperature-dependent photoluminescence (PL) measurements on these structures. It is found that the PL linewidth of the ILX is significantly larger than that of the excitons present in only the binary or only the ternary material layers. This is in contrast to our expectations.

## 2 Experimental details

### 2.1 Sample fabrication

**Fig. 2:**
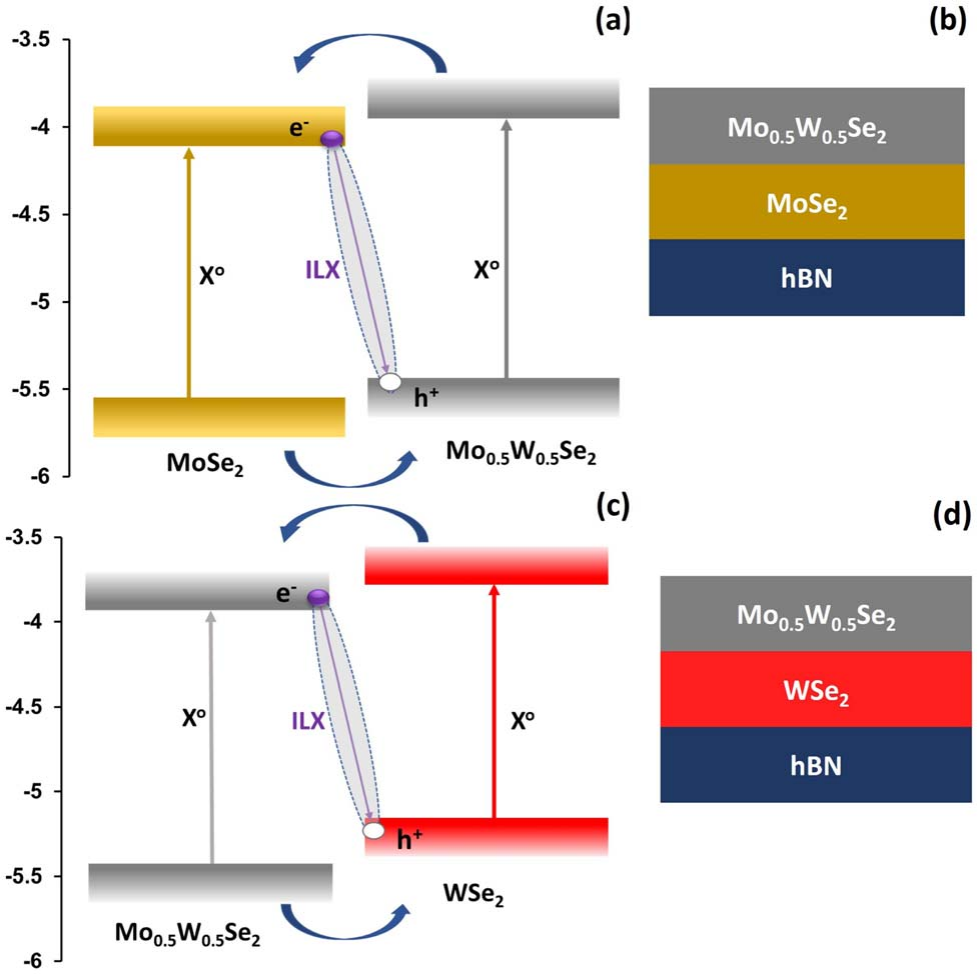
Schematic representation of estimated band alignment in binary–ternary heterostructures and structure of the fabricated heterostructure systems. Mo_0.5_W_0.5_Se_2_/MoSe_2_ is shown in (a and b) and Mo_0.5_W_0.5_Se_2_/WSe_2_ is shown in (c and d).

**Fig. 3:**
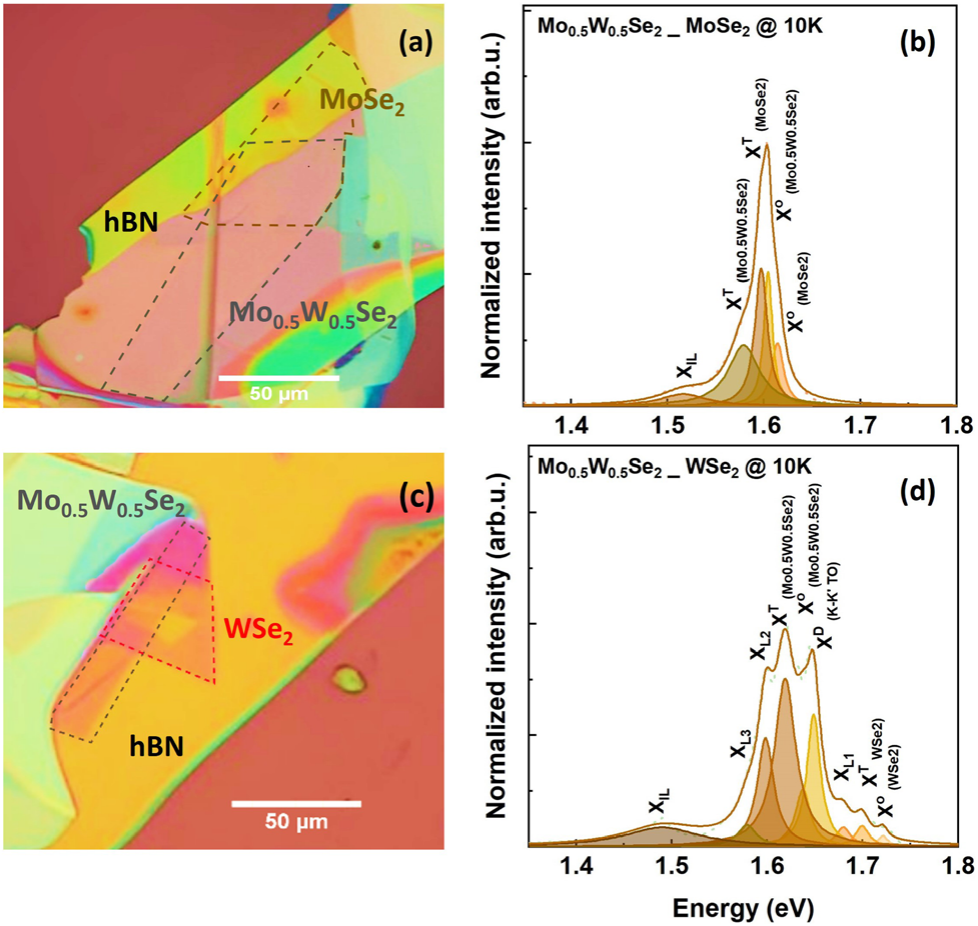
(a) Microscope image of the Mo_0.5_W_0.5_Se_2_/MoSe_2_ heterostructure under white light illumination (b) PL measurement on the Mo_0.5_W_0.5_Se_2_/MoSe_2_ sample collected from the HS spot at 10 K. (c) Microscope image of the Mo_0.5_W_0.5_Se_2_/WSe_2_ sample. (d) PL measurement on the Mo_0.5_W_0.5_Se_2_/WSe_2_ sample collected from the HS spot at 10 K.

The studied transition metal dichalcogenides (TMDs) heterostructures based on Mo_0.5_W_0.5_Se_2_/MoSe_2_ and Mo_0.5_W_0.5_Se_2_/WSe_2_ were fabricated through micro-mechanical exfoliation of commercially available bulk crystals. MoSe_2_ was obtained from, HQ Graphene (The Netherlands), while WSe_2_ and Mo_0.5_W_0.5_Se_2_ were obtained from 2D Semiconductors Inc (USA). The exfoliated flakes were transferred onto a polydimethylsiloxane (PDMS) gel placed on a glass slide for monolayer identification. We distinguished monolayers (MLs) from flakes of different thicknesses by their optical contrast. To ensure an atomically clean surface and homogenous dielectric environment for our heterostructure system, a few layers of hBN were transferred onto the target substrate (SiO_2_/Si). The identified TMDs monolayers were transferred on top of hBN flakes of several layers using the viscoelastic stamping technique. Subsequently, the PDMS was slightly lifted, leaving the MLs attached to the hBN flake. Laturia *et al.*^58^ found that 4–5 layers of hBN are usually sufficient for the dielectric constant to saturate in the out-of-plane direction. Thus, adding more layers beyond this does not significantly change the dielectric constant, as the properties start to resemble those of bulk hBN. Fig. 2(b) and (d) show a schematic representation of the prepared Mo_0.5_W_0.5_Se_2_/MoSe_2_ and Mo_0.5_W_0.5_Se_2_/WSe_2_ van der Waals heterostructures (vdW HSs), while Fig. 3(a) and (c) present the corresponding optical microscope images. During the fabrication and stacking of the monolayers, adsorbents and residuals may present at the interface between layers, potentially affecting the excitonic properties. To resolve this issue, a multi-stage sequential vacuum thermal annealing process was employed to minimise residuals and adsorbents. The as prepared samples were annealed under vacuum (∼10^−6^ mbar) at 150° for 4 hours. An additional annealing step at 300° for 4 hours was implemented to enhance the interlayer coupling between layers.

### 2.2 Optical characterization

Micro-photoluminescence (μ-PL) spectroscopy measurements were conducted using a home-built confocal microscope setup equipped with a nitrogen-cooled charge-coupled device (CCD) Si camera attached to the imaging monochromator (Princeton Instruments Acton SP2300). Additional details regarding the optical setup can be found in the ESI.† A frequency-doubled Nd–YAG continuous wave laser operating at 532 nm was employed as the optical excitation source. A set of mirrors directed the laser beam through a 70 : 30 beam splitter before being focused by a 40× microscope objective (NA = 0.6) into a tight Gaussian spot with a radius of approximately 2.5 μm. Furthermore, the laser power was fixed at 360 μW to prevent nonlinear processes such as exciton–exciton annihilation. The same microscope objective collected the emitted PL signal and directed it to the detection path. A 550 nm long-pass filter was placed in the detection path to filter out the laser from the PL signal. Furthermore, a CMOS camera and a flip mirror were incorporated into the detection path for optical control and imaging of the monolayers. For temperature-dependent measurements, the samples were mounted in liquid-helium flow microscopy cryostat (CryoVac) to control the temperature within the range from 10 to 300 K. To ensure seamless measurements, the microscope objective correction collar was set to a value of 1.5 mm to minimise aberrations caused by the cryostat's thick glass window (1.5 mm).

## 3 Results and discussion

An understanding of band offsets is crucial in the design of heterostructures employed in semiconductor devices. Band offsets influence the flow of charge carriers and are a key design parameter when selecting semiconducting materials for specific applications. Fig. 2 shows the energy level diagram and the structure of the two heterostructures studied. The energy level positions were adopted from Zhou *et al.*^59^ The upper part displays the situation for Mo_0.5_W_0.5_Se_2_/MoSe_2_. In this case, electrons are expected to be transferred from the ternary material into the binary material after optical excitation. In the lower part the situation for Mo_0.5_W_0.5_Se_2_/WSe_2_ is shown. In this case, the electrons will tunnel from the binary to the ternary material.

Microscope images of the two heterostructures produced are shown in Fig. 3(a) and (c). In Fig. 3(a) the MoSe_2_ and the Mo_0.5_W_0.5_Se_2_ monolayers are each outlined with dashed lines. The region of the Mo_0.5_W_0.5_Se_2_/MoSe_2_ heterostructure can be seen approximately centered in this image. Similarly, the Mo_0.5_W_0.5_Se_2_/WSe_2_ heterostructure can be seen slightly to the left of the center in Fig. 3(c). Fig. 3(b) and (d) show the low-temperature photoluminescence spectra of the two heterostructures. In both cases, a rich excitonic spectrum is observed. Detailed data analysis and the assignment of individual peaks to specific excitonic transitions are discussed in.^60^ Our assignment is consistent with other studies.^61–67^ The spectrum can be well described by a superposition of several excitonic peaks. The line shape assumed to fit the individual transitions is a Lorentz shape; following the approach from several reports^68–73^ in literature. Yet, with this approach, it is possible to determine the energetic position and spectral width of the individual excitonic transitions.

**Fig. 4:**
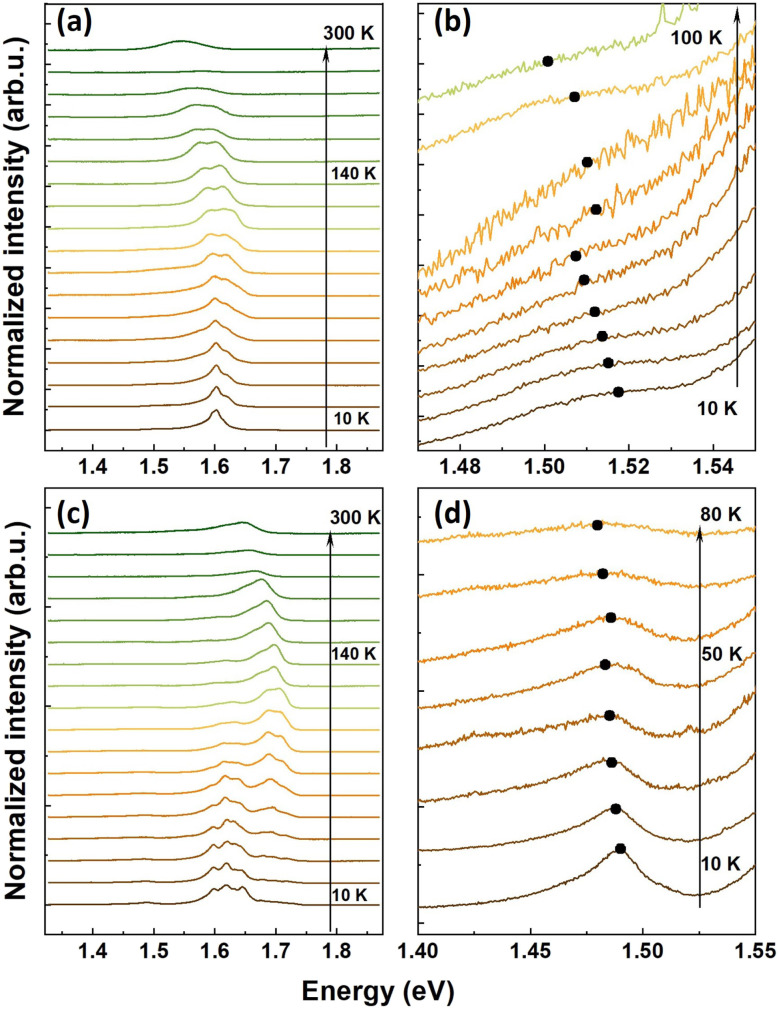
Photoluminescence of the (a) Mo_0.5_W_0.5_Se_2_/MoSe_2_ heterostructure as function of temperature. (b) Zoom of the spectral emission form the Mo_0.5_W_0.5_Se_2_/MoSe_2_ heterostructure in the ILX region. (c) Mo_0.5_W_0.5_Se_2_/WSe_2_ heterostructure. (d) Zoom of the spectral emission form the in Mo_0.5_W_0.5_Se_2_/WSe_2_ heterostructure in the ILX region.

**Fig. 5:**
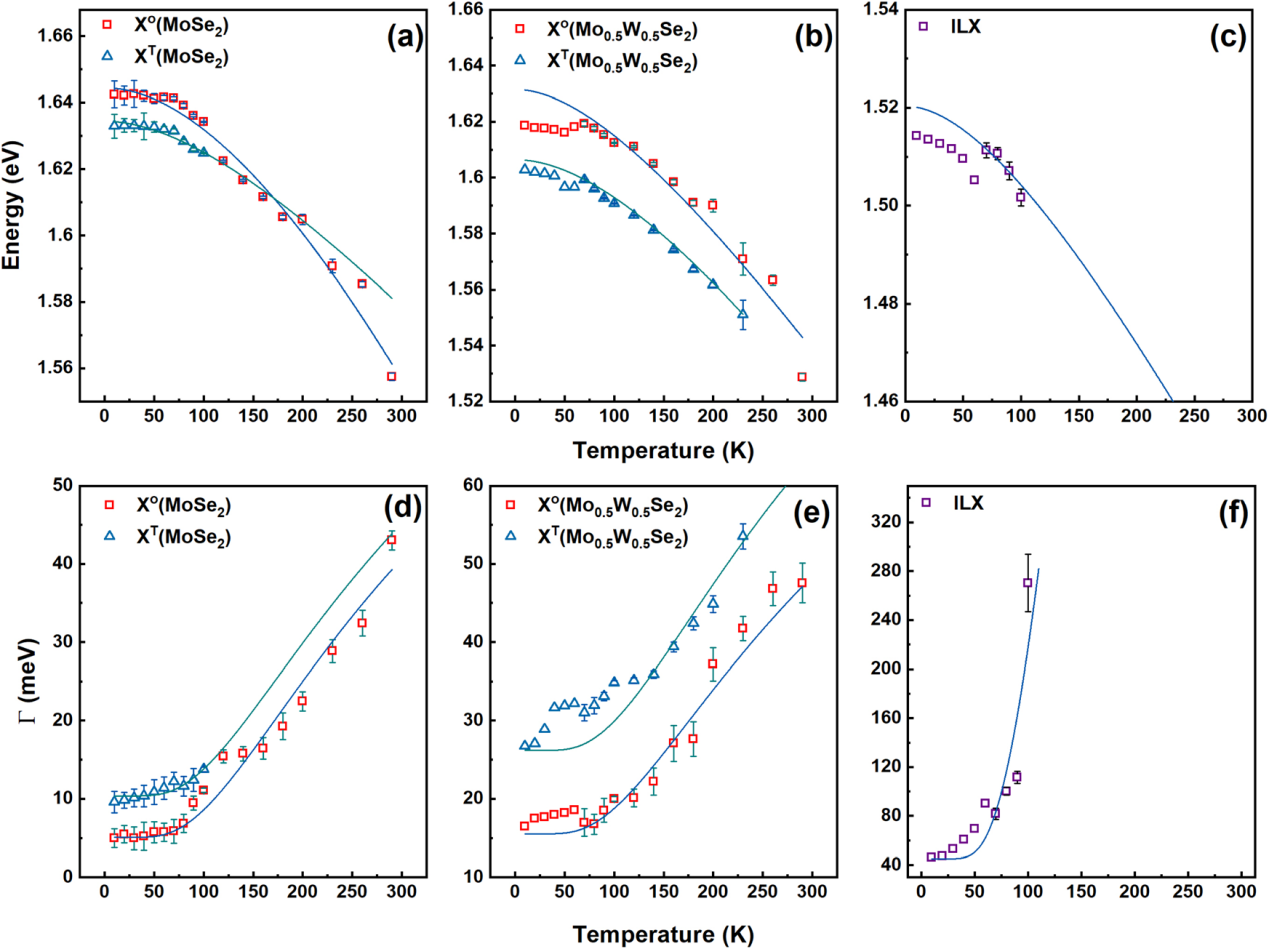
Photoluminescence peak position and linewidth as a function of temperature for the Mo_0.5_W_0.5_Se_2_/MoSe_2_ heterostructure. (a) Temperature-dependent MoSe_2_ PL peak energy of the exciton (red squares) and the trion (blue triangles). (b) Temperature-dependent Mo_0.5_W_0.5_Se_2_ PL peak energy of the exciton (red squares) and the trion (blue triangles). (c) Temperature-dependent PL peak energy of the interlayer exciton (violet squares). The solid lines represent the fits using the Varshni model. (d) Temperature-dependent MoSe_2_ linewidth of the exciton (red squares) and the trion (blue triangles). (e) Temperature-dependent Mo_0.5_W_0.5_Se_2_ linewidth of the exciton (red squares) and the trion (blue triangles). (f) Temperature-dependent interlayer exciton linewidth (violet squares). The solid lines represent the fits using the Rudin model.

**Fig. 6:**
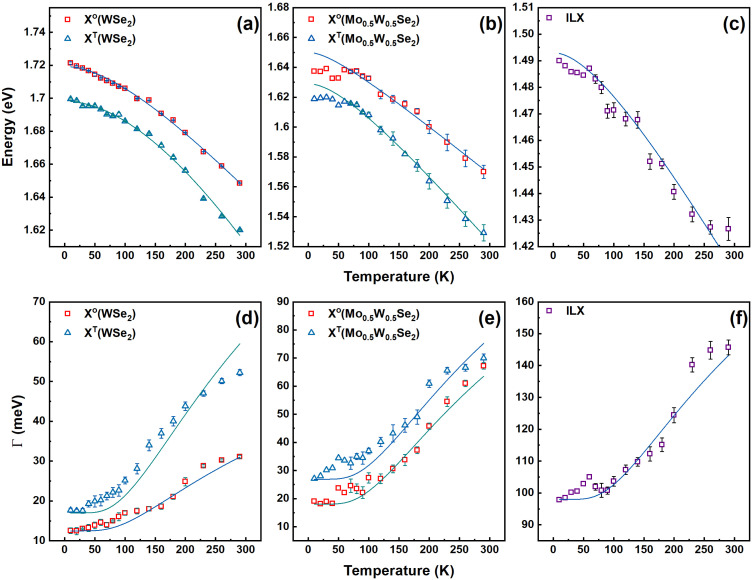
Photoluminescence peak position and linewidth as a function of temperature for the Mo_0.5_W_0.5_Se_2_/WSe_2_ heterostructure. (a) Temperature-dependent WSe_2_ PL peak energy of the exciton (red squares) and the trion (blue triangles). (b) Temperature-dependent Mo_0.5_W_0.5_Se_2_ PL peak energy of the exciton (red squares) and the trion (blue triangles). (c) Temperature-dependent PL peak energy of the interlayer exciton (violet squares). The solid lines represent the fits using the Varshni model. (d) Temperature-dependent WSe_2_ linewidth of the exciton (red squares) and the trion (blue triangles). (e) Temperature-dependent Mo_0.5_W_0.5_Se_2_ linewidth of the exciton (red squares) and the trion (blue triangles). (f) Temperature-dependent interlayer exciton linewidth (violet squares). The solid lines represent the fits using the Rudin model.

The luminescence signatures shift spectrally and experience a line broadening with increasing temperature. This is shown in Fig. 4(a) and (c) for the Mo_0.5_W_0.5_Se_2_/MoSe_2_ and Mo_0.5_W_0.5_Se_2_/WSe_2_ structure, respectively. Correspondingly, Fig. 4(b) and (d) show an enlarged view of the areas in which the luminescence of the interlayer exciton (ILX) is observed. The fits of the spectrum can be used to reveal changes in the energetic positions and linewidths of the individual excitonic peaks. The obtained values are plotted in Fig. 5 and 6. Fig. 5 shows in the upper part the energetic positions of the neutral exciton and the trion in the two 2D layers (MoSe_2_ and Mo_0.5_W_0.5_Se_2_). In addition, the energetic positions of the ILX, which is formed by charge carriers in both layers, is shown on the right. The corresponding linewidths of these excitonic transitions are shown in the lower part of the figure.

First, we discuss the shifting of the excitonic transition energies. The symbols represent the values obtained from the experimental data using the fits. The solid lines shown in Fig. 5 and 6 are based on Varshni^74^ and Rudin–Reinecke^75^ fitting functions for temperature-dependant PL peak position and linewidth respectively. A redshift with increasing temperature is observed for all excitonic transitions. This is the known behaviour, which can be explained by the Varshni function.^74^

In the case of the exciton in the ternary Mo_0.5_W_0.5_Se_2_, a clear “S-shape”^57,76–82^ is observed in the dependence of its energetic position on temperature. We attribute this effect primarily to alloy disorder. This observation is consistent with previous studies on 2D ternary materials.^41^ Also the ILX shows a clear “S-shape”; a sign of the presence of a pronounced disorder. Of interest, however, are the values determined for the associated linewidths. The line width of all excitonic transitions increases with temperature. This behaviour is also known and expected and can be described within the framework of Rudin and Reinecke model.^75^ More details about analysis, employed models and extracted fitting parameters can be found in the ESI.†

The linewidth of the neutral exciton in the binary material is in the range of 5 meV for low temperatures, *i.e.* it is relatively small. The linewidth increases significantly with temperature to a value of over 40 meV at room temperature. The values of the trionic transitions are slightly higher. In the ternary material, the linewidths of neutral excitons are significantly larger and amount to about 17 meV at low temperatures. We attribute this larger value compared to the binary material to alloy disorder.

It is interesting to note that the linewidth of the ILX is not between these two values, as we expected based on our initial considerations. Still, the linewidth of the ILX is considerably larger. At low temperatures, a value of 42 meV is determined. As the temperature increases, this value rises to above 100 meV. The same behaviour is observed for the second sample consisting of Mo_0.5_W_0.5_Se_2_ and WSe_2_. The corresponding data are shown in Fig. 6. For excitons and trions in the binary layer we do not observe an S-shape, but the excitons and trions in the ternary layer and the ILX show a hint of an S-shape. Again, the linewidth of the excitons and trions in the binary layer is the smallest while the ILX has the highest linewidth. The effect observed is therefore the same.

It is worth noting that very similar results were obtained by Nagler and coworkers on binary heterostructures of MoSe_2_ and WSe_2_ monolayers.^82^ Nagler *et al.* also found an S-shape in the energetic position of the ILX in temperature-dependent luminescence measurements. They, too, attributed this feature to disorder resulting from contaminations and inhomogeneities at the sample interfaces. They also reported linewidth values for excitons in the two binary layers and for the ILX. The low-temperature linewidth of the ILX was significantly larger than that of the excitons in the two binary layers, which agrees with our findings. Hence, the paper by Nagler *et al.* fully supports our conclusion.

Moreover, comparing Fig. 5b, c, 6b and c reveals that the S-shape is more pronounced in the WSe_2_-based heterostructure than in the MoSe_2_-based system. The effect of alloy disorder is the same for both structures since Mo_0.5_W_0.5_Se_2_ is the common monolayer. An energy scale of ∼2 meV is expected for *x* = 0.5, as reported by Baranovskii *et al.*^83^ Thus, the more prominent S-shape effect in the WSe_2_-based heterostructure can be attributed to more significant spatial interfacial inhomogeneity than the MoSe_2_ system. This results in a substantial additional contribution to the energy scale of the disorder potential.

The additional line broadening of the ILX observed here compared to the excitons in the 2D layers can only be caused by a dielectric disorder associated with the interface. The connection between the two layers of material is obviously not homogeneous, but exhibits inhomogeneities, *e.g.* small gaps or tiny impurities that have arisen during the sample production process. These findings show that the production of TMD heterostructures requires very clean work in order to minimise the disorder caused by the interface between the TMD monolayers. However, it is doubtful that this disorder can be completely avoided in TMD heterostructures produced by exfoliation.

## 4 Conclusions

In conclusion, we have performed temperature-dependent photoluminescence measurements on two binary–ternary heterolayers. The samples are composed of Mo_0.5_W_0.5_Se_2_ monolayers and monolayers of their binary counterparts, MoSe_2_ and WSe_2_. We can show that in both samples the PL linewidth of the ILX is significantly larger than that of the excitons existing only in the binary or only in the ternary material layers. This is in contrast to what one would generally expect. This leads us to the conclusion that additional disorder is present here. We attribute this line broadening to dielectric disorder caused by spatial inhomogeneity in the interface. Of course, it may be that encapsulating the heterostructure with hBN or producing the structure in a cleaner environment under an inert atmosphere can reduce these disorder effects, perhaps even significantly. However, no real sample will be completely free of disorder, so this effect will always be present, albeit reduced, even with a modified fabrication procedure. Thus, an understanding and a precise control of the interface quality and engineering at the nanoscale is vital to achieve high performance devices.

## Author contributions

Conceptualization, MK, HM, and MAA; methodology, MAA and EOE; validation, MK, HM, and MAA; formal analysis, MAA and EOE; investigation, MAA, EOE., and HM; resources, MK; data curation, MAA and EOE; writing—original draft preparation, MK, HM, and MAA; writing—review and editing, MAA, MK, and HM; visualization, MAA; supervision, MK and HM; project administration, MK and HM; and funding acquisition, MK and HM. All of the authors have read and agreed to the published version of the manuscript.

## Conflicts of interest

There are no conflicts to declare.

## Supplementary Material

NA-OLF-D4NA00786G-s001

## Data Availability

All the data and analysis results related to this study are presented in the main text as well as the ESI.†
