# Supplementary material for: Influence of interface dielectric disorder on interlayer excitons in mixed binary/ternary TMD heterostructures
Source: Nanoscale Adv. 2025 Jul 25;7(17):5337–45. doi: 10.1039/d4na00786g (PMC12290915; doi:10.1039/d4na00786g)
Supplement: NA-007-D4NA00786G-s001 [file NA-007-D4NA00786G-s001.pdf]

Supplementary Information (SI) for Nanoscale Advances.
This journal is © The Royal Society of Chemistry 2025

Supplementary Information

Influence of interface dielectric disorder on interlayer excitons in mixed binary/ternary TMD heterostructures†

Mohammed Adel Aly,[a,b,*±] Emmanuel Oghenevo Enakerakpor[a], Hilary Masenda,[a,c,||] and Martin Koch[a‡]

[a] Faculty of Physics and Materials Sciences Center, Philipps-Universität Marburg, 35032 Marburg, Germany;
[b] Department of Physics, Faculty of Science, Ain Shams University, 11566 Cairo, Egypt
[c] School of Physics, University of the Witwatersrand, 2050 Johannesburg, South Africa
‡ Corresponding Author:martin.koch@physik.uni-marburg.de
* Corresponding Author:mohammed.nouh@physik.uni-marburg.de
|| Corresponding Author:hilary.masenda@wits.ac.za
± Present address: Institute of Physics and Center for Nanotechnology, University of Münster, 48149 Münster, Germany

1 Optical Setup

The time-integrated micro-photoluminescence measurements were carried out using the set-up illustrated in Figure S1. An excitation laser light with a wavelength of 532 nm was focused onto the sample using a conventional confocal microscope setup. A 70:30 beam splitter and a 40× objective (with glass correction) were employed to focus the laser beam on the sample in a liquid Helium cryostat with a glass window. The PL signal from the sample was collected with the same objective and focused onto the spectrometer slit for the signal acquisition with a nitrogen-cooled CCD. White light was incorporated in the excitation path for sample imaging using a removable lens and flip mirror coupled with a CMOS camera.

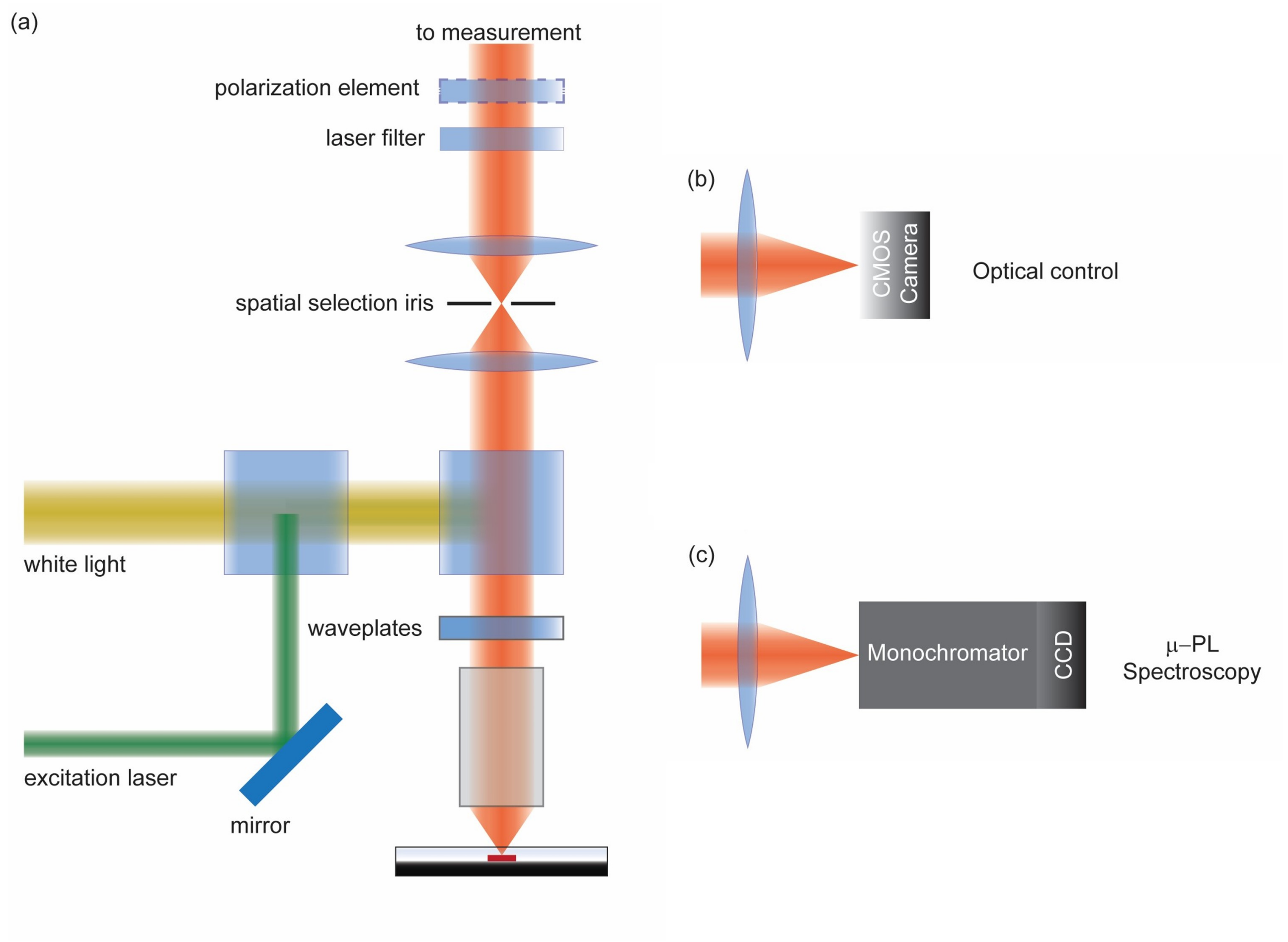

Fig. S1 Schematic diagram of the low-temperature micro-photoluminescence setup.

2 Peak position Analysis

The obtained fitting parameters of the excitonic peaks according to fit based on the Varshni model[1] for both heterostructure systems are listed in table 1 and 2. The respective formula is:

$$E_{\mathrm{op}}(T) = E_{\mathrm{op}}(0) - \frac{\alpha_v T^2}{T+\beta}, \tag{1}$$

where $E_{\mathrm{op}}(0)$ represents the optical bandgap at zero temperature, α_v and β are fitting parameters related to the temperature-dependent dilatation of the lattice and to the Debye temperature, respectively. This equation is based on the theoretical studies of the electron-phonon interactions causing the dependence of $E_g(T)$, which were carried out already in 1950s[2–6].

Table 1 Extracted parameters obtained from the fits of the PL peak energy by Eq. (1) for $MoSe_2$ based sample.

$Mo_{0.5}W_{0.5}Se_2$ / $MoSe_2$					
	$MoSe_2$		$Mo_{0.5}W_{0.5}Se_2$		ILX
**Parameter**	X^0	X^-	X^0	X^-	ILX
$E_{op}(0)$ [eV]	1.648	1.638	1.632	1.605	1.52
α_v [$\times$ 10^{-4} eV K^{-1}]	5.7	5.74	5.57		4.92
β [K]	300		240.7	241	200

Table 2 Extracted parameters obtained from the fits of the PL peak energy by Eq. (1) for WSe_2 based sample.

$Mo_{0.5}W_{0.5}Se_2$ / WSe_2					
	WSe_2		$Mo_{0.5}W_{0.5}Se_2$		ILX
**Parameter**	X^0	X^-	X^0	X^-	ILX
$E_{op}(0)$ [eV]	1.722	1.6983	1.65	1.63	1.493
α_v [$\times$ 10^{-4} eV K^{-1}]	4.23		5.5	6.44	4.34
β [K]	179.13		225	220	173

3 Linewidth Analysis

The obtained fit parameters of the excitonic peaks based on the Rudin-Reinecke model[7] for both heterostructure systems are listed in table 3 and 4. The respective formula is:

$$\Gamma_{PL}(T) = \Gamma_{PL}(0) + \gamma_{LA} T + \Gamma_{LO} \left[\frac{1}{\exp(E_{LO}/kT) - 1} \right]. \quad (2)$$

The term $\Gamma_{PL}(0)$ for the PL-linewidth at 0 K is supposed to contain all the effects for the line broadening at $T = 0$ K and, hence, includes the inhomogeneous contributions caused by the disorder potential. The coefficient γ_{LA} denotes the exciton-acoustic phonon coupling strength, while Γ_{LO} stems from the interaction with longitudinal optical (LO) phonons.

Table 3 Extracted parameters for the linewidth obtained from the Rudin-Reinecke model for $MoSe_2$ based sample.

$Mo_{0.5}W_{0.5}Se_2$ / $MoSe_2$					
	$MoSe_2$		$Mo_{0.5}W_{0.5}Se_2$		ILX
**Parameter**	X^0	X^-	X^0	X^-	ILX
$\Gamma_{PL}(0)$ [meV]	5.02	9.95	13.96	25	40
Γ_{LO} [meV]	41.53	52.1	40.13	42.2	2000
E_{LO} [meV]			30		

Table 4 Extracted parameters for the linewidth obtained from the Rudin-Reinecke model for WSe_2 based sample.

$Mo_{0.5}W_{0.5}Se_2$ /WSe_2					
	WSe_2		$Mo_{0.5}W_{0.5}Se_2$		ILX
**Parameter**	X^0	X^-	X^0	X^-	ILX
$\Gamma_{PL}(0)$ [meV]	12.49	17	26.9	18.04	97.81
Γ_{LO} [meV]	24.5	50.08	59.45	59	60.6
E_{LO} [meV]			30		

4 Visualization

The schematic depiction of the heterostructure in the Table of Contents is based on crystallographic data provided by the Materials Project[8–10] and drawn by Mercury software version 4[11] and the VESTA software version 3[12].

References

1 Y. P. Varshni, Physica, 1967, **34**, 149–154.
2 T. Muto and S. Oyama, Progress of Theoretical Physics, 1950, **5**, 833–843.
3 H. Y. Fan, Physical Review, 1951, **82**, 900–905.
4 E. Antončúk, Czechoslovak Journal of Physics, 1955, **5**, 449–461.
5 H. D. Vasileff, Physical Review, 1957, **105**, 441–446.
6 E. N. Adams, Physical Review, 1957, **107**, 671.
7 S. Rudin and T. L. Reinecke, Physical Review B, 1990, **41**, 3017–3027.
8 Materials Project, Material System: MP-1821, https://next-gen.materialsproject.org/materials/mp-1821, Accessed: November 27, 2024.
9 Materials Project, Material System: MP-1634, https://next-gen.materialsproject.org/materials/mp-1634, Accessed: November 27, 2024.
10 Materials Project, Material System: MP-984, https://next-gen.materialsproject.org/materials/mp-984, Accessed: November 27, 2024.
11 C. F. Macrae, I. Sovago, S. J. Cottrell, P. T. A. Galek, P. McCabe, E. Pidcock, M. Platings, G. P. Shields, J. S. Stevens, M. Towler and P. A. Wood, Journal of Applied Crystallography, 2020, **53**, 226–235.
12 K. Momma and F. Izumi, Journal of Applied Crystallography, 2011, **44**, 1272–1276.
